# Non-neoplastic bulky mediastinal mass presentation in an adolescent patient: a case report

**DOI:** 10.1186/1752-1947-7-233

**Published:** 2013-10-02

**Authors:** Paula Fraiman Blatyta, Claudio Carneiro Borba, Ligia Reis de Queiroz, Raphael Salles Scortegagna de Medeiros, Fabiana Gomes de Campos, Israel Bendit

**Affiliations:** 1 Disciplina de Hematologia e Hemoterapia da Faculdade de Medicina da Universidade de Sao Paulo, Av. Dr. Eneas de Carvalho Aguiar 155, primeiro andar, sala 30, Sao Paulo, CEP 05403-000, Brazil; 2 Hospital Samaritano de São Paulo, São Paulo, Brazil; 3 Instituto de Tratamento do Câncer Infantil, Universidade de Sao Paulo, São Paulo, Brazil; 4 Grupo Fleury de Medicina Diagnóstica, São Paulo, Brazil; 5 Hospital A.C. Camargo, São Paulo, Brazil

## Abstract

**Introduction:**

Mediastinal masses in pediatric patients are very heterogeneous in origin and etiology. In the first decade of life, 70% of the mediastinal masses are benign whereas malignant tumors are more frequent in the second decade of life. Among the mediastinal masses, lymph nodes are the most common involved structures and could be enlarged due to a lymphoma, leukemia, metastatic disease, or due to infectious diseases as sarcoidosis, tuberculosis and others.

**Case presentation:**

We report a case of a 13-year-old Caucasian girl who came to the emergency room with a history of intermittent fever, weight loss and night sweating for at least 1 month. A radiologic image work-up presented an anterior and posterior mediastinal mass. The ^18^F-fluorodeoxyglucose positron emission tomography presented a high maximum standard uptake value, which directed our decision for mediastinal biopsy for diagnostic elucidation. Histologic examination described the mass as granulomatous tuberculosis. The patient was treated with anti-tuberculosis therapy and developed a full clinical recovery.

**Conclusions:**

The present case report demonstrates that a bulky mediastinal lymphadenopathy detected on ^18^F-fluorodeoxyglucose positron emission tomography is not always a malignant lesion, and in countries where tuberculosis is endemic, this etiology should not be forgotten during clinical investigations. There is a need for more accurate cut-off values for this technology; meanwhile, the further investigation of patients with bulky mediastinal masses with procedures such as the open biopsy is indispensable.

## Introduction

Mediastinal masses in children can be caused by the expansion of many organs that are contained in this thoracic compartment, such as the thymus gland, the thoracic portion of the esophagus and trachea, the great vessels, the heart, lymph nodes, fat and nerves. Non-vascular mediastinal masses can derive from all these structures and represent many conditions, such as congenital anomalies, benign and malignant neoplasms and infection [1]. Lymphomas account for 13% of all pediatric cancers and are the most common cause of a mediastinal mass in children; almost half of the children with non-Hodgkin and two thirds of the children with Hodgkin lymphoma present with anterior or middle mediastinal masses [2].

We describe an adolescent girl with a mediastinal mass and persistent fever that became a diagnostic challenge due to conflicting results among imaging resources.

## Case presentation

A 13-year-old Caucasian girl was admitted as an in-patient to our Pediatric Ward. During the first consult in the Pediatric Emergency Room, the family described a history of intermittent fever up to 39°C during the past month. She had no history of pruritus, but described some weight loss and night sweating. The physical examination showed some paleness and a discrete rash in her upper body. The chest X-ray showed a right-sided mediastinal enlargement and a left parahilar bulging. At this time, a complete blood count revealed levels of hemoglobin at 10.4g/dL, a whole blood count of 8.2×10^9^/L and platelets of 511×10^9^/L. She also had a slightly elevated lactate dehydrogenase of 582U/L and C-reactive protein of 5mg/dL. Thoracic computed tomography (CT) described a bulky mass within all mediastinal compartments, extending to the left pulmonary hilum and displacing the vascular mediastinal structure without invasion. It was described as an extensive agglomerate of lymph nodes. The abdominal CT described an increased number of normal-sized lymph nodes in the mesenteric root. Considering these results, the hypothesis of a neoplastic disorder or an inflammatory disease, such as histoplasmosis or paracoccidioidomicosis was considered.

**Figure 1:**
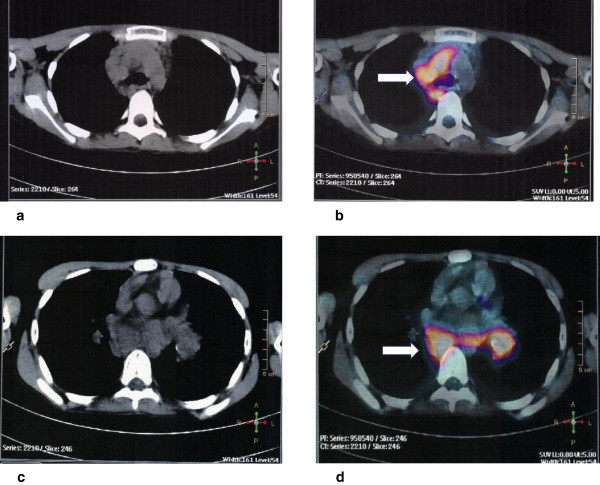
**(a-c) Axial contrast-enhanced computed tomography image of the mediastinal region, and (b-d) fused transverse **^**18**^**F-fluorodeoxyglucose-positron emission tomography-computed tomography image at the mediastinal region where the arrow is pointed there was an increased **^**18**^**F-fluorodeoxyglucose uptake in the anterior and posterior mediastinum as well as in the left peribronchial nodes.**

**Figure 2:**
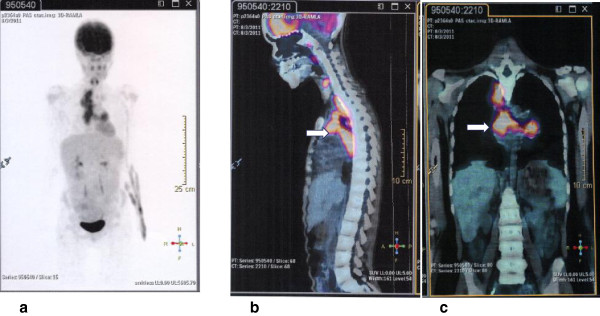
**(a) Anterior view of a maximum intensity projection **^**18**^**F-fluorodeoxyglucose positron emission tomography, (b-c) Sagittal and coronal fused **^**18**^**F-fluorodeoxyglucose-positron emission tomography-computed tomography images of intense hypermetabolism in the anterior and posterior mediastinal masses (arrows).**

**Figure 3:**
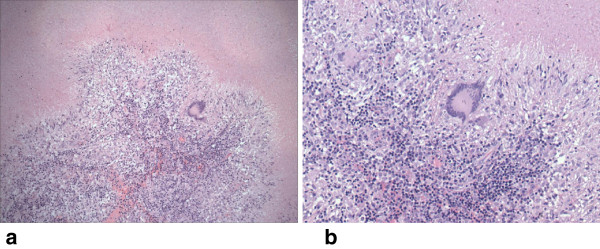
The image shows lack of the architecture of lymphoid tissue and depletion of lymphocytes which were replaced by large areas of caseous necrosis (a), and granulomatous chronic inflammation accompanied with Langhans-type multinucleate giant cells (b) (photomicrographs obtained from hematoxylin and eosin-stained histologic sections at ×100 and ×200μm).

As fever persisted, serology for cytomegalovirus, Epstein–Barr virus, toxoplasmosis, aspergillosis, histoplasmosis, brucellosis, *Chlamydophila pneumoniae*, rickettsiosis, and paracoccidioidomicosis were requested and the results were negative. Meanwhile, her parents informed us that the child had regular contact with an aunt who works as a health agent in a public health clinic that treats patients with tuberculosis (TB). After this information was elicited, three samples of acid-fast bacilli sputum-stained smears were ordered, and all the tests were negative; simultaneously, a tuberculin skin test was ordered and resulted positive 72 hours after inoculation (15mm of induration). About the same time a positron emission tomography (PET) scan was performed in order to clarify the diagnosis. It showed multiple areas of anomalous concentration of ^18^F-fluorodeoxyglucose (^18^F-FDG) in the thoracic region, corresponding to coalescent lymph nodes in the right anterior and posterior mediastinal and left peribronchial clusters of lymph nodes, with standard uptake value (SUV) between 5.6 and 6.3 (Figures 1 and 2). These findings were considered highly suspicious for neoplastic disease, and a tissue biopsy was recommended. The patient underwent a mediastinal biopsy through a thoracotomy, which enabled the recovery of a fragment of the posterior mediastinal mass. The fragment was described as yellowish, wrinkled and with a caseous appearance. Hematoxylin and eosin-stained sections of lymph nodes revealed distinct and well-formed epithelioid cell granulomas (Figure 3a). There was complete effacement of lymph node architecture by these granulomas. Variation in the size of the granulomas was observed as well as wide areas of caseation and compressed granulomas with fibrosis. Perinodal and perivascular granulomas were seen. The capsule in all biopsies was intact and showed thickening. Pericapsular fibrosis and fibrosis within the granuloma were noted. Giant cells of the Langhans type within the granulomas were seen frequently. Typical caseation necrosis in the center of the granulomas was observed (Figure 3b). A meticulous search for bacilli using Ziehl–Neelsen stain was performed and scanty and only occasional bacilli were found in the epithelioid cells and caseating necrotic material (not shown). Based on these findings, the final diagnosis was tuberculous lymphadenitis.

The TB treatment prescribed was rifampicin and isoniazid, and the child showed gain of weight and fever recrudescence during the first 6 months on medication. The follow-up chest CT after 1 year of therapy showed an improvement in the size of her mediastinal lymph nodes.

## Discussion

The reported case highlights the difficult diagnosis of TB in children. It is speculated that TB in children may represent 10 to 15% of the total number of infected people around the world [3,4], but its diagnosis has been neglected in this population due to atypical radiological features in children, the difficulty in obtaining material for tests (sputum) and the fact that cultures have an extremely low positivity rate. There is also a common perception among health providers that children do not transmit TB, which has been proved to be a misconception [5]. It is also well known that TB can mimic many other pathologies in children, another obstacle in the recognition of TB’s magnitude among this population worldwide [2,4]. In pediatric patients, the most common symptoms associated with the disease are pulmonary parenchymal disease and thoracic adenopathy, accounting for 60 to 80% of cases [6]. The main hypothesis for our patient was a malignant disease (lymphoma) considering the length of fever and weight loss, and the impressive image of mediastinal enlargement. The imaging resources contributed to the misinterpretation of the symptoms, as the PET scan showed a high maximum SUV, asymmetry and an enlarged node size in CT, findings suggestive of neoplastic disease [7,8]. Nevertheless, a PET scan has a lower specificity (false positive results) for metabolically active infectious or inflammatory lesions, which can accumulate FDG (for example, rheumatoid nodules, tuberculous granulomata, fungal granulomata, pneumonia, empyema, drug toxicity, and sarcoidosis) [9,10]. That is why some studies suggest the SUV value that indicates malignancies should be increased in countries where TB and sarcoidosis are endemic [6], in order to reduce the rate of false-positive results and the indication of unnecessary procedures. Our patient went through a thoracotomy to obtain a tissue biopsy, as the PET diagnosis was divergent from the tuberculin skin test results and it was necessary to exclude a malignancy.

## Conclusions

This case report raises the alert that TB should be considered a differential diagnosis in many pediatric clinical backgrounds because it is still a prevalent and insidious burden globally. There is a need for more accurate cut-off values for the PET technology; meanwhile, the further investigation of patients with bulky mediastinal masses with procedures such as the open biopsy is indispensable.

## Consent

Written informed consent was obtained from the patient’s legal guardian for publication of this case report and accompanying images. A copy of the written consent is available for review by the Editor-in-Chief of this journal.

## Competing interests

The authors declare that they have no competing interests.

## Authors’ contributions

IB and PFB are the first authors and designed the study, analyzed the data, and wrote the manuscript. CCB and LRQ were the patient’s medical doctors during inward hospitalization, analyzed the data, and reviewed the manuscript. RSSM was responsible for the pathology study and revised the manuscript. FG analyzed the data and helped to edit the manuscript. All authors have read and approved the final version of this manuscript.

## Authors’ information

Israel Bendit is supported by Fundação Maria Cecília de Souto Vidigal.
